# Pathological and clinical outcomes following neoadjuvant dual HER2 therapy for early‐stage breast cancer: An Australian institutional real‐world experience

**DOI:** 10.1002/cam4.7325

**Published:** 2024-06-20

**Authors:** Sathya Narayanan, Nicholas K. Ngui, Ben Kinchington, Joseph Do Woong Choi, Thomas Michael D. Hughes, Josie Rutovitz, Csilla Hasovits, Kazi J. Nahar, Senarath Edirimanne, Gavin Marx

**Affiliations:** ^1^ Cancer Services, Sydney Adventist Hospital Sydney New South Wales Australia; ^2^ Macquarie University Sydney New South Wales Australia; ^3^ Northern Haematology and Oncology Group Sydney Adventist Hospital Sydney New South Wales Australia; ^4^ School of Medicine and Psychology The Australian National University Canberra Australia; ^5^ Northern Surgical Oncology Sydney Adventist Hospital Sydney Australia; ^6^ University of Sydney Sydney New South Wales Australia; ^7^ Melanoma Institute Australia Sydney New South Wales Australia

## Abstract

**Aim:**

There has been significant progress made in developing novel targeted therapies in the neoadjuvant setting for non‐metastatic HER2‐positive breast cancer, which may be used in combination with conventional chemotherapy to optimise pathological responses at surgery. However, these therapies, particularly the chemotherapeutic components, may portend significant and long‐lasting toxicity. Hence, de‐escalation of treatment intensity has been an area of interest and was evaluated in the phase II NeoSphere study. Herein, we report the real‐world pathological and survival outcomes from neoadjuvant taxane and dual HER2 blockade recorded at our centre.

**Methods:**

This was a retrospective cohort study of patients receiving neoadjuvant pertuzumab, trastuzumab and taxane chemotherapy for non‐metastatic HER2‐positive breast cancer at a single centre in Sydney, Australia. We collected data pertaining to baseline demographic characteristics, pathological response rates, post‐surgical prescribing patterns and also undertook survival analyses for invasive disease‐free survival (iDFS) as well as exploratory analyses for correlations between pre‐specified clinicopathologic factors and pathological response at surgery.

**Results:**

Our population was largely similar at baseline to the NeoSphere study. 71 patients were included in the final analysis. 61% achieved a pathological complete response (pCR). Three patients received conventional chemotherapy in the adjuvant setting. 92% of included patients were alive and disease‐free at 3 years of follow‐up. Only 3 events of recurrence or death were recorded at a median follow‐up of 32 months. No significant difference in iDFS was noted between patients achieving pCR and those with residual disease at surgery.

**Conclusion:**

This study demonstrates that de‐escalated adjuvant treatment for HER2‐positive early breast cancer achieved favourable pathological and long‐term outcomes comparable to large trials, some utilising more intensive chemotherapeutic components.

## 1 INTRODUCTION

Breast cancer is a heterogeneous entity necessitating an individualised approach to therapy. Amplification or overexpression of the HER2 oncogene is identified in 15% of breast cancer patients.^1^ This phenomenon promotes pathways which drive angiogenesis, increased cell proliferation and resistance to apoptosis.^2^ The resulting increased biological activity facilitates a greater sensitivity to neoadjuvant chemotherapy, with greater rates of pathological complete response (pCR) at surgery.^3^ Response rates may be improved further with the addition of targeted therapies such as trastuzumab and pertuzumab.^4^

A common treatment approach for non‐metastatic HER2‐positive breast cancer is the administration of systemic therapy comprised of cytotoxic chemotherapy with HER2 directed agents in the neoadjuvant setting. The addition of pertuzumab to trastuzumab has been investigated in the neoadjuvant context, following demonstration of benefit in the metastatic setting.^5^ The phase II NeoSphere trial randomised HER2‐positive patients to various neoadjuvant regimens involving combinations of taxane chemotherapy, pertuzumab and trastuzumab. The pCR rate was 46%, the best observed amongst the arms of this study, in patients receiving trastuzumab, pertuzumab and docetaxel.^4^

A variety of chemotherapeutic regimens have been tested in the neoadjuvant setting for early HER2‐positive breast cancer. Given the considerable associated toxicity both in the short and long term, de‐escalating chemotherapy intensity before and after surgery has been a recent area of interest. The ongoing international multicentre phase II DECRESCENDO trial examines the administration of neoadjuvant taxane with dual HER2 blockade, with the omission of conventional adjuvant chemotherapy.^6^

In 2019, our group published an Australian first case series examining pCR rates in patients treated at our institution with neoadjuvant taxane and dual HER2 inhibition with trastuzumab and pertuzumab. We demonstrated greater pCR rates compared to NeoSphere (68% vs. 46%) with reasonable tolerability.^7^ Herein, we aim to build on our previous study regarding pathological response rates in a larger cohort, and also describe survival outcomes up to 4 years following treatment, noting that the vast majority of our patients did not receive further adjuvant chemotherapy, particularly those who achieved pCR. Pathological response is a controversial surrogate endpoint for long‐term outcomes beyond the individual patient level.^8^ Based on the findings of the NeoSphere study and self‐funded access to dual targeted therapy, we have been administering de‐escalated systemic therapy comprised of a taxane alone with dual HER2 blockade since 2018, without administration of other agents such as carboplatin or anthracyclines in the neoadjuvant setting. As such, our real‐world data capturing short‐term survival outcomes, rather than just pathological response, may help support chemotherapy de‐escalation as an appropriate and safe approach, as currently being evaluated in the ongoing DECRESCENDO trial.

## 2 MATERIALS AND METHODS

This was a retrospective cohort study of patients receiving neoadjuvant pertuzumab, trastuzumab and taxane chemotherapy for early or locally advanced HER2‐positive breast cancer treated at the Sydney Adventist Hospital. The included patients were treated from December 2016 to June 2022. As described previously, all patients at our institution with HER2‐positive early or locally‐advanced breast cancer were treated with single‐agent taxane, trastuzumab and pertuzumab. Inclusion criteria were: (1) Confirmed HER2‐positive breast cancer (defined as IHC 3+ or ISH positive regardless of IHC) treated with neoadjuvant taxane, pertuzumab and trastuzumab therapy (2) staged as operable (T1‐3, N0‐1, M0) or locally advanced (T2‐3, N2‐3, M0; T4a‐c, any N, M0), (3) no previous anti‐cancer therapy for HER2‐positive breast cancer and (4) aged 18 or more. The exclusion criteria included confirmed metastatic disease.

Patients received a loading dose of 8 mg/kg of trastuzumab, 840 mg of pertuzumab and taxane chemotherapy. Subsequent trastuzumab and pertuzumab dosing was 6 mg/kg and 420 mg every 3 weeks respectively. All patients received weekly paclitaxel at a standard dose of 80 mg/m^2^, unless clinical or biologic parameters necessitated an upfront dose reduction. Patients were scheduled to receive 12 cycles of weekly paclitaxel and four cycles of pertuzumab and trastuzumab prior to surgery. Post‐surgical prescribing patterns following pathological response evaluation at surgery were described. The primary objective was to describe the neoadjuvant and adjuvant prescribing regimens, and pathological response rates. Secondary objectives were to describe short‐term outcomes of disease‐free survival (DFS) and analyse any associations with pre‐specified clinicopathological factors and pCR. pCR was defined as no residual invasive disease in the breast and axilla.^3^

### 2.1 Ethics approval

Protocols were reviewed and approved by an independent ethics committee according to institutional requirements; an exemption was granted pursuant to the National Statement on Ethical Conduct in Human Research (2007), updated 2018; sections 5.1.22 and 5.1.23 (Adventist HealthCare Limited HREC).

### 2.2 Instruments, measures and procedures

Clinicopathological data were retrospectively collected for all sequentially treated patients receiving this regimen from the iCIMS (Innovative Clinical Information Management Systems) and SanCare (SanCare Electronic Medical Record software, Sydney Adventist Hospital) databases. The patients were treated at the Sydney Adventist Hospital between December 2016 and June 2022. The data collected included demographics, pathological characteristics (primarily ER/PR status) and T stage, nodal involvement, details of treatment regimens administered, post‐surgery prescribing patterns and adverse events.

### 2.3 Statistical analysis

Baseline characteristics and exploratory analyses were populated using SPSS version 29.0.0.0 (IBM). Figures were generated using jamovi version 2.3. Exploratory analyses were conducted regarding predictors of pathological response at surgery. A binomial logistic regression model was implemented to explore for associations between the pre‐stipulated independent variables of age, nodal status, T stage, HER2 IHC (2+ or 3+), ECOG performance status, oestrogen and progesterone receptor status and the total cumulative dose of paclitaxel administered with pathological response at surgery, recorded as a dichotomous variable (pathological complete response vs. residual disease).

## 3 RESULTS

The baseline characteristics are shown in Table 1. 71 patients were included in our final analysis. The median age of included patients was 57 (range: 35–83). All our patients had a performance status of 0 or 1. The majority of our patients were T1 or T2, with only 9% having T3 disease. 50% of patients with known lymph node status had confirmed nodal involvement. 54% of patients were ER positive and 41% were both ER and PR negative on core biopsy prior to neoadjuvant therapy.

**TABLE 1:** Baseline characteristics and interventions post neoadjuvant therapy.

Characteristic	Study population *n* = 71 (%)
Age (years), median (range)	57, 35–83
ECOG Performance Status, *n* (%)	
0	30 (42.3)
1	41 (57.7)
T stage of tumour pre‐treatment, *n* (% of available data)	
T1	30 (42.3)
T2	35 (49.3)
T3	6 (8.5)
Nodal involvement at diagnosis, *n* (% of available data)	
Involved	35 (49.3)
Uninvolved	36 (50.7)
Hormone receptor status on pre‐treatment core biopsy, *n* (% of available data)	
ER positive, PR positive	29 (40.8)
ER positive, PR negative	9 (12.7)
ER negative, PR positive	4 (5.6)
ER negative, PR negative	29 (40.8)
Surgical intervention, *n* (% of available data)	
WLE and SLNB &/or TAD	36 (52.2)
Mastectomy and SLNB &/or TAD	12 (17.4)
WLE and axillary clearance	7 (10.1)
Mastectomy & axillary clearance	14 (20.3)
None^a^	1
Unknown	1
Adjuvant therapy administered, *n* (% of available data)	
FEC 100	1 (1.4)
Trastuzumab	53 (74.6)
Trastuzumab emtansine	13 (18.3)
Trastuzumab and pertuzumab	1 (1.4)
Trastuzumab and FEC	2 (2.8)
Nil administered^a^	1 (1.4)

Abbreviations: SLNB, sentinel lymph node biopsy; TAD, targeted axillary dissection.

^a^ One patient died during neoadjuvant therapy and hence did not undergo surgery or adjuvant therapy.

With regard to neoadjuvant treatment administered, 69% of patients received all 12 recommended cycles of paclitaxel, with almost 90% receiving at least 11. All patients (except for one patient who died during the course of neoadjuvant therapy) received all four cycles of recommended pertuzumab. 64 patients (90%) received four cycles of neoadjuvant trastuzumab. Five received more than four cycles of trastuzumab, while two received fewer. Adjuvant therapy varied within our cohort, but notably 94% of patients did not receive adjuvant conventional chemotherapy. The vast majority (around 75%) of patients received trastuzumab alone to complete the prescribed 17 cycles (1 year) of treatment. 13 (18%) received adjuvant TDM‐1. This comprised 50% of the 26 patients with residual disease at surgery. Patients who did receive adjuvant conventional chemotherapy all had an RCB‐II at surgery.

Thirty‐six patients (52%) underwent a wide local excision and sentinel lymph node biopsy (SLNB) &/or targeted axillary dissection (TAD). 12 patients (17%) underwent a mastectomy, SLNB &/or TAD, with 14 (20%) undergoing mastectomy and axillary clearance. Seven patients (10%) had a wide local excision and axillary clearance. One patient died during neoadjuvant therapy and their pathologic and survival outcomes were censored from analysis. Forty‐three patients (61%) achieved a pCR, seven patients (10%) achieved a Residual Cancer Burden (RCB) RCB‐I, 19 patients (27%) achieved an RCB‐II, while no patients were identified to have RCB‐III disease at surgery. Pathological response data were missing for two patients.

A binomial regression was performed to evaluate the effects of age, T stage, nodal involvement, ECOG performance status, oestrogen and progesterone receptor status, HER2 IHC status (2+ vs. 3+) and the total cumulative dose of taxane on pathological response. The logistic regression model was statistically significant χ^2^(10) = 20.079, *p* = 0.029. The model explained 34.2% of the variance of observed pathological response (Nagelkerke R^2^) and correctly classified 73.9% of cases. Of the independent variables included, none contributed significantly to the predictive value of the model at an alpha threshold of 0.05. Only HER2 IHC approached the threshold for statistical significance (*p* = 0.087). If significant, patients with IHC 3+ were 14.1 times more likely to obtain a pCR compared to patients with IHC 2+. Pre‐treatment characteristics in relation to pCR rates are summarised in Table 2.

**TABLE 2:** Pre‐treatment characteristics and pCR rates.

Characteristic	*N* (%)	pCR (%)	*p‐value* (regression model)
Age			0.145
<50	21 (29.6)	13 (61.9)	
50–74	43 (59.2)	29 (67.4)	
>75	6 (9.9)	1 (16.7)	
Unknown/missing	1 (1.4)	N/A	
Nodal status			0.471
Positive	34 (47.9)	19 (55.9)	
Negative	36 (50.7)	24 (66.7)	
Unknown/missing	1 (1.4)	N/A	
Oestrogen receptor status (on pre‐treatment biopsy)			0.267
ER positive	37 (52.1)	19 (51.4)	
ER negative	33 (46.5)	24 (72.7)	
Unknown/missing	1 (1.4)	N/A	
Progesterone receptor status (on pre‐treatment biopsy)			0.496
PR positive	32 (45.1)	16 (50.0)	
PR negative	38 (53.5)	27 (71.1)	
Unknown/missing	1 (1.4)	N/A	
ECOG			0.363
0	29 (40.8)	21 (72.4)	
1	41 (57.7)	22 (53.7)	
Unknown/missing	1 (1.4)	N/A	
HER2 IHC (on pre‐treatment biopsy)			0.087
2+	9 (12.7)	1 (1.1)	
3+	60 (84.5)	41 (68.3)	
Unknown/missing	2 (2.8)	N/A	
T stage			0.662
T1	29 (40.8)	19 (65.5)	
T2	35 (49.3)	21 (60.0)	
T3	6 (8.5)	3 (50.0)	
Unknown/missing	1 (1.4)	N/A	

Abbreviation: pCR, pathological complete response.

**FIGURE 1:**
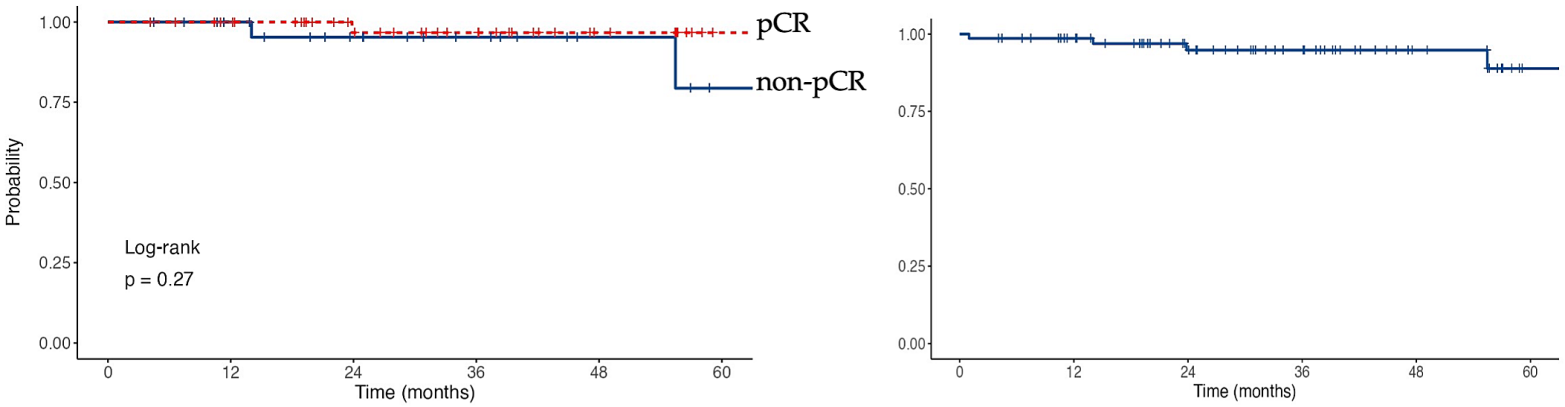
Kaplan–Meier curves for invasive disease‐free survival by pathological response at surgery (left) and in whole population (right).

Patients were followed up from commencement of first treatment for a median of 32 months (range 1.0–80.1 months). Of the included patients, only three patients experienced recurrent disease, one of whom had a pCR, with one patient assessed as RCB‐I and another being RCB‐II at surgery. One patient died only 6 weeks after starting treatment secondary to diarrhoea, complicated by gastrointestinal bleeding and pneumonitis. Of the 62 patients who were followed up for 12 months or more, 61 (98%) were alive and disease‐free at 12 months. Of the 48 patients who were followed up for 24 months or more, 45 (94%) were alive and disease‐free. At 3 and 4 years respectively, 92% of the 36 patients and 86% of the 21 patients who were followed up for these time intervals were alive and disease‐free. One recurrence event occurred after 4 years at 55.5 months of follow‐up. The invasive disease‐free survival (iDFS) by pathological response (left) and in the entire cohort (right) omitting the one patient who died before surgery and therefore without pathological response data at each follow‐up timepoint is summarised in Figure 1. There was no significant difference in iDFS in our population between patients achieving a pCR at surgery and those with residual disease (*p =* 0.27). However, we do note a higher percentage of patients being alive and disease‐free at each timepoint between patients with a pCR at surgery compared to those with residual disease, admittedly with very few recurrence events noted at each timepoint.

## 4 DISCUSSION

In our Australian institutional cohort of 71 patients with HER2‐positive non‐metastatic breast cancer, 61% achieved a pCR with neoadjuvant taxane and dual HER2 inhibition. This compares favourably to the findings of the NeoSphere trial, with 46% of patients achieving a pCR.^4^ We report only three events of recurrence over a median follow‐up of 32 months. There was one treatment‐related death despite the therapy being otherwise well‐tolerated.

There is a need for further characterisation of pre‐treatment correlates with pathological response in early HER2‐positive breast cancer, particularly in the era of dual HER2 inhibition and the potential for de‐escalation of treatment in appropriately selected patients. With regards to HER2 staining by IHC, a study with a subgroup of patients receiving neoadjuvant dual HER2 inhibition with chemotherapy demonstrated a significant association between HER2 IHC 3+ and improved pCR rates.^9^ Our study suggests that cancers with a stronger HER2 IHC expression are more likely to have a better pathologic response, although this discrepancy did not meet statistical significance. There is evidence demonstrating an association between hormone receptor negativity and improved pCR rates.^3^ This was also noted in the NeoSphere study, with the ER positive group achieving a pCR rate of 63.2% compared to 26% in the ER positive group.^4^ Contrary to prior literature, we did not find a significant difference in pCR rates by oestrogen receptor status in our sample. Numerically, however, we did note that 73% of patients with ER negative disease obtained a pCR compared to 51% in the ER positive subgroup.

Another important question is the utility of pCR as a valid surrogate endpoint for long‐term outcomes in HER2‐positive early breast cancer (EBC). As described previously, pathological response at surgery is associated with disease‐free and overall survival at the individual patient level, whereby patients who achieve a pCR have better disease‐free and overall survival compared to those who do not. This correlation has been most strongly associated with triple‐negative breast cancer, followed by HER2‐positive breast cancer. The first work to investigate this association was a pooled analysis including over 12,000 patients from 12 separate trials by the Collaborative Trials in Neoadjuvant Breast Cancer (CTNeoBC) group. Unfortunately, despite demonstrating that pCR was associated with event free survival (EFS), the degree of treatment‐associated impact on pCR rates and corresponding amelioration of EFS was not established.^3^ Similarly, another meta‐regression involving 29 trials of neoadjuvant therapy for EBC demonstrated only a weak association between the quantitative improvement of pathological response with therapy compared to equivalent impact on long‐term outcomes.^10^

However, a more recent meta‐analysis including over 27,000 patients with early breast cancer, with over 5000 patients being HER2‐positive, demonstrated a significantly better EFS (HR 0.31; 95% PI: 0.24–0.39) for patients attaining a pCR in the entire group, with particularly strong associations noted for the HER2‐positive subgroup (HR 0.32; 0.21–0.47).^11^ In addition, the neoadjuvant platform I‐SPY2 trial concluded that for HER2‐positive patients with early breast cancer, pCR at surgery was associated with a risk reduction in recurrence of 80%.^12^ Hence, although pCR attainment does have proven prognostic importance, it remains a controversial surrogate endpoint. More specifically, it is a strong patient‐level surrogate, but a weak trial‐level surrogate, in that in randomised trials, the impact of treatment on the surrogate does not consistently and reliably predict the impact of treatment on the final endpoint.^13^ As such, further data is required to validate pCR as a truly valid surrogate endpoint in HER2‐positive EBC. Our study may help strengthen the surrogacy of pathological response, given the relatively high observed pCR rates and favourable short‐term survival outcomes. There was no statistically significant difference (*p* = 0.27) in iDFS between patients achieving a pCR at surgery compared to those without, although our study was underpowered.

Our pCR rates compared favourably to the NeoSphere study. In addition, as seen in Table 3, apart from the unprecedented pathological response rates seen in the WSG‐ADAPT study,^14^ our results are largely superior to other published results. It is important to note that our population included both hormone‐receptor positive and negative patients in comparison to the WSG‐ADAPT study which included only hormone‐receptor negative patients who might be expected to have higher pCR rates based on existing evidence. Further evaluation of tailoring adjuvant therapy following de‐escalated neoadjuvant treatment based on pathological responses is required in the prospective setting, and large trials are currently underway, namely the DECRESCENDO and CompassHER2 studies.^6^, ^15^

**TABLE 3:** pCR rates with neoadjuvant taxane and dual HER2 inhibition—comparative studies.

Study	*n*	Hormone receptor status of included patients	pCR rates
Current study: Narayanan et al., 2024	71	Both included	61% all population 73% ER negative 51% ER positive
DAPHNE^16^	95	Both included	57%
WSG‐ADAPT^14^		HR negative only	ypT0/N0 79%, ypTisN0 90.5%
I‐SPY2^12^	44	Both included	54%
NEOPETRA (real‐world, Spanish)^17^	243	Both included	59.3%
Ma et al., 2022, (real‐world, Chinese)^18^	63	Both included	53.1%
PeRSIA (real‐world, Australian)^19^	95	Both included	64.1%
Sharma et al., 2022^20^	71	Both included	56%

We recorded three events at 2 years, with a resultant two‐year iDFS of 94%. The NeoSphere study reported long‐term outcomes at 5 years and described a DFS of 84% for the group receiving taxane and dual HER2 inhibition, with subsequent provision of three cycles of adjuvant FEC chemotherapy.^21^ The WSG‐ADAPT study reported a 5‐year relapse‐free survival of 98%, with adjuvant chemotherapy administered in patients not achieving a pCR and left to the investigator's discretion in patients achieving a pCR.^14^ The APHINITY trial, evaluating the addition of pertuzumab to trastuzumab and chemotherapy in the adjuvant setting identified a 3‐year iDFS of 94.1% in the group receiving pertuzumab, with 78% of patients receiving anthracycline‐containing adjuvant chemotherapy.^22^ Other large trials, namely TRYPHAENA, BERENICE, TRAIN‐2 and KRISTINE, utilising a multiplicity of chemotherapeutic backbones for dual HER2 inhibition, including either carboplatin or an anthracycline, 3‐year DFS rates ranged from 92% to 94%.^23^, ^24^, ^25^, ^26^ Evaluation of long‐term outcomes with neoadjuvant taxane and dual HER2 inhibition in real‐world settings is still lacking in the published literature. O'Shaughnessy et al. (2021) reported recurrence rates in 217 patients in patients with a pCR post neoadjuvant dual HER2 inhibition and recorded a 4‐year iDFS of 90%.^27^ A Dutch series describing long‐term outcomes for 453 patients receiving neoadjuvant dual HER2 inhibition as part of the TRAIN‐2 study reported a 5‐year BCSS of 95%. However, disparate chemotherapeutic backbones were administered in combination with targeted therapy, uniformly more intensive than that administered in our study.^25^ In any case, our long‐term outcomes in a real‐world setting are comparable to existing literature from large trials.

The vast majority of our patients received no conventional chemotherapy in the adjuvant setting, with only three patients receiving adjuvant FEC. In accordance with findings from the KATHERINE trial, our patients with residual disease at surgery since 2020 received adjuvant trastuzumab emtansine (TDM‐1), while those with a pCR completed their year of trastuzumab monotherapy.^22^ This medication was listed on the Pharmaceutical Benefits Scheme in Australia for this indication in 2020.^28^ As such, only 50% of patients with residual disease at surgery received adjuvant TDM‐1. One patient received pertuzumab in the adjuvant setting in combination with trastuzumab. Data around adjuvant pertuzumab remains sparse; however, the PEONY trial suggested an EFS, DFS and OS benefit with pertuzumab (vs. placebo) administered in the adjuvant setting in combination with trastuzumab for a total of 1 year of targeted therapy. Of note, three cycles of FEC chemotherapy were also administered in the adjuvant setting in this study.^29^

There are notable limitations to this study. Our retrospective study sample is small, involving 71 patients managed at a single institution in Australia. As such, no significant difference in our limited cohort between iDFS between pCR and non‐pCR subgroups was found. Moreover, our sample size analysis of short‐term outcomes is limited, with data available for only 48 patients at 2 years. The authors intend to report longer term survival outcomes in a future study. Our exploratory analyses regarding predictors of pathological response should be interpreted with caution, given the limited sample size of our study and the multiple comparisons made. In addition, we note the potential pitfalls of cross‐trial comparisons in our discussion. Despite these limitations, this is the largest Australian study reporting both pathologic and survival outcomes in patients treated with neoadjuvant dual HER2 therapy with de‐escalation of adjuvant chemotherapy.

## 5 CONCLUSION

This study demonstrates that de‐escalated adjuvant treatment for HER2‐positive early breast cancer achieved favourable pathological and long‐term outcomes comparable to large trials, some utilising more intensive chemotherapeutic components. Furthermore, our demonstration of high pCR rates with dual HER2 therapy with favourable short‐term outcomes provides real‐world evidence to suggest that pCR appears to be a potential surrogate of DFS.

## AUTHOR CONTRIBUTIONS

**Sathya Narayanan:** Conceptualization (equal); data curation (lead); formal analysis (lead); investigation (lead); methodology (lead); supervision (supporting); validation (lead); visualization (lead); writing – original draft (lead); writing – review and editing (lead). **Nicholas K. Ngui:** Conceptualization (equal); resources (equal); supervision (equal); visualization (equal); writing – review and editing (lead). **Ben Kinchington:** Data curation (lead). **Joseph Do Woong Choi:** Data curation (supporting); writing – review and editing (supporting). **Thomas Michael D. Hughes:** Conceptualization (equal); resources (equal); writing – review and editing (supporting). **Josie Rutovitz:** Writing – review and editing (supporting). **Csilla Hasovits:** Resources (equal); writing – review and editing (supporting). **Kazi J. Nahar:** Writing – review and editing (supporting). **Senarath Edirimanne:** Writing – review and editing (supporting). **Gavin Marx:** Conceptualization (equal); methodology (equal); resources (equal); supervision (equal); writing – review and editing (equal).

## FUNDING INFORMATION

This research received no external funding.

## CONFLICT OF INTEREST STATEMENT

The authors have no conflicts of interest to declare.

## INSTITUTIONAL REVIEW BOARD STATEMENT

The study was performed in accordance with the ethical principles outlined in the Declaration of Helsinki and that are consistent with International Council for Harmonisation (ICH)/Good Clinical Practice (GCP) and applicable regulatory requirements. Protocols were reviewed and approved by an independent ethics committee (Sydney Adventist Healthcare Limited) having been granted an exemption pursuant to the National Statement on Ethical Conduct in Human Research (2007), updated 2018; sections 5.1.22 and 5.1.23.

## Data Availability

The data that support the findings of this study are available from the corresponding author upon reasonable request.
